# “HIV Changed My Life Forever”: An Illustrative Case of a Sub-Saharan African Migrant Woman Living with HIV in Belgium

**DOI:** 10.3390/tropicalmed2020012

**Published:** 2017-05-23

**Authors:** Agnes Ebotabe Arrey, Johan Bilsen, Patrick Lacor, Reginald Deschepper

**Affiliations:** 1 Department of Public Health, Faculty of Medicine and Pharmacy, Vrije Universiteit Brussel, 1090 Brussels, Belgium; johan.bilsen@vub.ac.be (J.B.); rdeschep@vub.ac.be (R.D.); 2 Department of Internal Medicine and Infectious Diseases-AIDS Reference Center, Universitair Ziekenhuis Brussel, 1090 Brussels, Belgium; patrick.lacor@uzbrussel.be

**Keywords:** HIV, AIDS, choice-disabled, depression, mental health, migrant woman, vulnerability

## Abstract

Living with HIV and AIDS changes everything for people diagnosed with HIV and it can be the most difficult experience in life. Like most people who have chronic diseases, these individuals have to deal with living a normal and quality life. Globally, more women (51%) than men are HIV positive. The main aim of this paper was to describe a sub-Saharan African migrant woman’s lived experience, and also to use the individual’s story to raise questions about the larger context after a HIV diagnosis. A qualitative study consisting of a personal story of a HIV-infected sub-Saharan African living in Belgium was conducted. Data were analysed using thematic analysis. The main themes that emerged from the data included relational risks, personal transformation and the search for normality, anxiety, depression, fear of stigma, societal gender norms, and support. The participant reported that marriage was no guarantee of staying HIV-free, especially in a male-dominant culture. This case further illustrates that married and unmarried African women are often at high risk of HIV and also informs us how HIV could spread, not only because of cultural practices but also because of individual behaviour and responses to everyday life situations. The participant also emphasized that she is faced with physical and mental health problems that are typical of people living with HIV. The vulnerability of sub-Saharan African women to HIV infection and their precarious health-related environments wherever they happen to be is further elucidated by this case.

## 1. Introduction

Globally, an estimated 36.7 million people are living with HIV, of which about 51% are women [1]. Living with HIV and AIDS changes everything for people diagnosed with HIV and it can be the most difficult experience in life [2,3]. Like most people with chronic diseases, these individuals have to deal with living a normal and quality life. The diagnosis of any chronic illness tends to disrupt or dissolve the basic essence of living [4] and the chronically ill person becomes aware of the complications that may hinder his or her ability to live in a world that is becoming more individualistic [5]. HIV is still far from being wiped out, as evidenced by the number of new infections registered every year [6].

The physical and mental suffering surrounding HIV challenges health promotion [7], especially among certain marginalized groups like migrants [8], women [9], sexual orientation minorities, ethnic minorities and intravenous drug users, with increased risk of new HIV transmission. In addition to HIV, there are many socio-economic and cultural factors such as poverty, fragile family structures, stigma and discrimination that people from low-resource and high prevalence regions like sub-Saharan Africa have to wrestle with in order to maintain quality of life wherever they live [10,11].

Migration has been held responsible for the spread of HIV because of its complex relationship with health [12,13,14]. The process of migration may create certain vulnerabilities like access to healthcare, employment, economic progress, education, housing and legal status [15,16]. Some groups of migrants may be disproportionately affected by HIV, and it is essential to understand migrant health in order to understand the factors that foster vulnerability to HIV. HIV among migrants in the European Union, especially those from sub-Saharan Africa (SSA), is a public health concern and thus, it is of prime importance that this group of migrants be targeted when taking measures to increase HIV awareness and prevention [17].

Gender dimension is relevant and key to understanding the persistence of HIV. Women represent the majority of HIV persons [1], especially where unprotected heterosexual contacts are the main mode of HIV transmission [18]. Many women living with HIV, especially from male-dominant cultures in many parts of the world, are very reluctant to disclose their status for fear of unfair treatment in their communities, and losing their loved ones, home, property and jobs [19,20]. Three interconnected factors place gender issues at the centre of the HIV pandemic among sub-Saharan Africans wherever they are found. The psychological, cultural and socio-economic factors account for the differences in risk and vulnerability between men and women [21].

Stigma, discrimination and gender inequality are consequences of the HIV pandemic but can also act as a driving force [22]. Stigma has been summarised as “the phenomenon whereby an individual with an attribute, which is deeply discredited by his or her society, is rejected as a result of the attribute” [23]. Stigmatizing attitudes can have adverse effects, including difficulties in personal relationships, disruption of vocational, professional and educational goals and, crucially, delay in seeking help. Stigma surrounding HIV has a particularly heavy impact on women and the stigma experienced by women has remained a cause of high HIV prevalence over the last three decades in the world, sub-Saharan Africa and Belgium [24]. Increasingly, women and adolescent girls are affected disproportionately and it is vital to integrate gender into HIV programmes to curb this phenomenon, which may eventually reduce the spread of the disease [25]. Unfortunately, both women and men are put at risk of infection as a result of societal gender ideals and norms, exposing all to the phenomenon of stigma and discrimination. The stigma and discrimination surrounding HIV has a particularly heavy impact on women from the time of diagnosis, at whatever age, throughout the illness trajectory [26,27].

Despite the free, available and accessible combination antiretroviral therapy for HIV patients in most EU countries [28,29], most migrants living with HIV often relate to the fear of diagnosis [30], disclosure and being rejected by others and manifest the feelings of hopelessness, depression and social distancing [31]. This results in many HIV migrants coping with their diagnosis through (non)-disclosure of HIV-positive status, isolation and avoidance, support and empathy and alcohol/substance abuse [11,32,33,34]. Many HIV-infected migrants live with mental health issues, including depression, anxiety and stress that may greatly impact treatment and care by their reluctance to seek care, and risky behaviours. According to many studies, HIV remains a much stigmatized illness in the society that influences the process of disclosure and often necessitates secrecy about illness in the families, communities and healthcare settings [34,35,36,37].

The main aim of this paper was to describe a SSA migrant woman’s lived experience with HIV, and also to use the individual’s story to raise questions about the larger context after an HIV diagnosis. The uniqueness of this case is the longevity with HIV as a result of free, accessible and available antiretroviral therapy (ART), and the biopsychosocial and spiritual issues experienced by this woman and most SSA migrants living with HIV.

## 2. Materials and Methods

### 2.1. Ethical Approval

All procedures were approved by the Ethics Committees of the Universitair Ziekenhuis Brussel (Approval number B.U.N. 143201215911) and the Institutional Review Board (IRB) of the Institute of Tropical Medicine, Antwerp, Belgium (Approval number IRB/AB/ac/141). The approved informed consent form made provisions for the participant to include name, contact address and signature if she agreed to participate. The confidentiality of the participant was respected by removing all identifying elements from data. The participant was free to withdraw from study at any time. There was no financial compensation. Following the confidentiality section on the approved informed consent form, the participant could opt not to give contact addresses or withdraw from the study without fear of repercussion on treatment and care. All identifiers were removed from the text. Neither the participant nor the researcher was exposed to unnecessary risk. Due to the sensitive nature of the study, the audio-recordings and transcripts are stored and data cannot be made public in terms of ethical restrictions protecting participant confidentiality.

### 2.2. Study Design and Sample

This qualitative descriptive paper is based on a distinctive case from a wider study that was conducted between April 2013 and December 2014 among 44 SSA migrant women living and aging with HIV in Belgium. The case includes the narrative of a woman diagnosed with HIV about 27 years ago. This case was selected because of certain uniqueness including the duration, physical and mental challenges living with HIV. We refer to our participant simply as “P” to protect her identity. The use of narratives have been posited as the preferred method for researching people with chronic illnesses such as mental disorders, cancer, diabetes, asthma and HIV [38,39,40].

### 2.3. Data Collection

A semi-structured interview and observation of the participant were used to collect data on how a HIV-positive diagnosis can change the life of an infected woman. The interview lasted for 35 minutes.

### 2.4. P’s Storyline

I got married when I was about 20 years old and we had a baby. We wanted to have another baby and I made this desire known to my aunt, a nurse, who advised us to take the HIV test. I had heard of HIV when I was still living in Africa in the early eighties. The testing was voluntary. My husband, my aunt and I went to the hospital to get the HIV test done. To my greatest surprise my husband and I were diagnosed HIV positive. That was 27 years ago. I was not prepared for the diagnosis and I was in complete shock. I thought of many things. I saw my desire to have more children dwindle away. I did not stop crying. I just needed to be alone and started crying. I was so depressed that I thought of ending my life several times. I thought of my daughter that I would not see grow up, the life that I could not have and the marriage that was the cause of my illness. I thought of death, death, but finally, I became aware of the fact that I could live with the disease.

I thank God and I am happy that I could receive good treatment. We were fortunate enough and came regularly to Belgium for treatment and care before the civil war that killed many people in my country. The outbreak of the civil war was the reason for my immigrating to Belgium. Had I stayed back home, it would have been impossible for me to travel to Belgium and continue receiving care. I would probably be dead by now. My condition did not necessitate taking medications until I moved to Belgium.

My husband confessed to having had extra-marital sexual relations and asked for forgiveness. I am quite sure he knew who he had had sexual contacts with that resulted in the infection. I don’t know if he knew that he had contracted HIV before we took the test. We were not ill before testing and have not been ill since our diagnoses because of the good medical care we receive in Belgium. I would have probably been dead if I was not taking the HIV treatment. We try to adhere to our treatment strictly and continue having a normal life. Our love helped us to accept the diagnoses and incorporate HIV into our daily lives. Moreover, we had an infant and we were determined to fight and ensure a better future for her. My fear of transmitting the HIV virus through childbirth made me decide not to have another child.

I still blame my husband for infecting me with HIV because while growing up, I had desired to have many children. My treating doctor assured me that I could have a child without the child being infected but I did not want to experiment with a child. At that time I had no confidence in medical and scientific claims that a child could be born free of the virus even in Europe. I saw many people die from AIDS in Africa and was not sure that I would survive. I feel hurt whenever I think that I could have had more children and have lived through this very badly. However, as time went on, we spent a lot of time discussing the management of the disease and read about new advances in scientific research on finding a cure and new medications, until four years ago when we started experiencing tension in our relationship.

At the onset of the armed conflict in early 1990s (name of country has been withheld for confidentiality reasons) we moved to Belgium for safety and where I continued receiving treatment. We go to the clinic and are treated without fear that our HIV status will be disclosed to people who know us. I don’t pay for the HIV treatment and care I receive. I get free treatment and care. The insurance reimburses my treatment and care. I am very satisfied with the healthcare I receive. I would have probably been dead if not for the medications and great support from the HIV care providers. I am happy to be in Belgium and receiving treatment and care.

I stayed permanently while my husband travelled between my home country and Belgium because we have a business which he has to manage. He is absent most of the time and this absence has caused a lot of strain in our relationship. The tension between us is not related to the HIV disease but to living apart and his way of life. I felt so lonely and because of this loneliness, I resorted to drinking heavily, which made me more depressed. I am aware that too much alcohol is not good when someone is on HIV treatment but I could not control my urge to continue drinking, the only way I believed I could mediate my life with HIV. I accepted detoxification treatment to help me stop drinking. Alcohol abuse became another illness that I had to fight.

I try to look at the positive side of things when I realized that I had to live with this illness all my life. Many people have succumbed to this illness. I am lucky to be alive and I thank God for giving me another chance. Thus, I must not abuse this opportunity. I take my medications and I feel well. I can do anything I want to and my illness has not rendered me handicapped. I try to move forward and live normally but there are always periods of depression. I take care of myself, my health, my hygiene and what I eat. However, it is difficult to live with this disease. I have high cholesterol, maybe because of the medications I’m taking. I also have problems with my bones. I live permanently with these problems. I have to pay attention to everything I do. To me, the most difficult thing about living with this illness is not having more children.

I disclosed my HIV-positive status to my parents and sisters but not to friends. I also informed my husband’s mother about our infection. I don’t know who my husband might have told. The support I get from my family helps me to continue living a normal life. I am not in any HIV support group in Belgium. I feel that joining such groups will make me more depressed, so I stay away. People with HIV see life with HIV differently. I want to avoid being in a situation where I will feel more depressed because of another person’s stressors. Some people are very negative and I don’t want that to have any negative effect on me. In the beginning I used to feel very uncomfortable seeing billboards and posters on HIV everywhere but I got used to the fact that the public needs to be sensitized and in my opinion, I think the Belgian government is doing great in trying to increase public awareness of the seriousness of HIV infection.

I do blame men for the propagation of this disease. My advice to women and others who are not infected with HIV is to prevent themselves from contracting the disease. The uninfected have a wonderful opportunity to remain vigilant at all times. They should try not to reject those who are infected with HIV. To those who are infected with HIV, I encourage them to continue taking their medications, take care of themselves, use condoms to protect themselves from reinfections that can further weaken their immune systems and render them susceptible to infections. They should also take measures to protect their children and others from being infected with the virus.

I am a devout Catholic Christian and my relationship with God has not changed. I believe this illness has consolidated my relationship with God. This illness has re-enforced my faith in God. I was religious before contracting HIV and I believe that my belief in God has helped me cope with life as an HIV patient and the deception I experienced in my marital life.

In my opinion our culture has a role to play in the spread of HIV. The serious nature of the disease was not taken into consideration when the virus started spreading back in the early 1980s. The disease and mode of transmission was minimized because people are used to fatalism. They claimed that death would come anyhow and anywhere (war, hunger, malnutrition, accidents), not necessarily through AIDS. I also think that poverty has contributed to the spread of HIV. Young girls have relationships with rich older men who may be infected and subsequently, these men infect their wives and the vicious circle continues. I find this dishonest. I once confronted my husband to find out if he took precautions when he had extra-marital sexual relations so as not to infect other women. He simply looked at me and said nothing. In addition to poverty, ignorance of HIV status by many people, multiple sexual partners, lack of care in relationships and the general way of life that permits male dominance have contributed to the spread of HIV among the African communities. Lack of good hygiene and sanitation can also expose people to the HIV virus.

Prior to my alcohol abuse treatment, I tried to contribute the fight against HIV by helping hospitalized AIDS women and children orphaned by AIDS in my country (name withheld). Sometimes, these women were hospitalized and they had to stay in the hospital with their young children because their husbands could not care for the kids. Despite the fact that these children were not sick, they slept under their mothers’ hospital beds. In fact it’s disheartening there.

Every 3 months I travelled back to Africa to check on the conditions of the women and children I tried to help. My actions consisted of buying meal vouchers from the hospital, enough for multiple meals for 20 persons for a period of 3 months. The hospital sells meal vouchers reserved for people living with AIDS at reduced rates. During my stay, I paid regular visits to these women to encourage and empathize with them. While back in Belgium, I collected used clothing and shoes to send back to the women and children I am helping. I sensitized European and African women about HIV without disclosing my HIV positive status to them. With my alcohol problem, I could no longer organize assistance to these HIV-infected women in my country.

The lessons I have learnt from living with HIV are to appreciate good health, humility and carefulness. There are precautions that I have to take when preparing food for myself and towards others. Another lesson I have learnt is to live each day as if it is the last, giving the best of yourself. I try doing this every day. I continue my journey and I have confidence for the future.

Actually, I’m the first among the HIV patients in Belgium. I am still alive after 27 years. This horrible illness does not shout when it comes. It is there and unfortunately it has been very difficult with the absence of my husband and the loneliness, but I have to survive. I thank God for this.

### 2.5. Observation During Hospitalisation

P was hospitalized at the university teaching hospital a few months after being interviewed. The researcher visited P after being informed by P that she was admitted. The purpose of the visit was to provide support, interact socially with P in order to observe and understand her. This involved the observation of working practices of ward nurses and the participant. Notes were taken as to what was observed, heard and seen.

### 2.6. Data Analysis

A single case was selected for analysis to provide a detailed description of a SSA migrant woman diagnosed with HIV. Thematic analysis was conducted to identify themes rooted in the basic principles of grounded theory techniques (coding, constant comparison) [41] involving three phases: open, axial and selective coding. A rational for using some basic grounded theory techniques is that this technique can be used for any form of data collection. The tape-recorded interview was transcribed by the first author. The transcript from the interview and notes from the observations were read by the first author to identify patterns of words or statements related to the focus of the study. In the open coding phase themes were identified as they emerged from the data, in line with the study objective. The transcription was coded into key words and phrases to construct categories grounded on data [42,43]. During the axial coding phase, relationship, context, and strategies used to manage the phenomenon (HIV) and the consequences, and excerpts that could better explain the basic themes were extracted. Categories were then formed and analysed to identify themes reflecting the findings within data, a procedure known as selective coding.

The research team frequently reflected on coding decisions during the analysis process. This reflexivity allowed researchers to bracket their biases, knowledge, experience, and personal feelings to minimize their influence on the analysis process. Themes related to the topic were identified by constantly comparing new themes grounded in data. Saturation was reached when no new information was retrieved from data. The first and fourth authors read and analysed this interview and discussed their findings to obtain consistency, validity and credibility. The use of thematic analysis was important in the identification of new themes that recurred in the participant’s story and that could eventually lead to universal observations.

## 3. Results

Major themes that emerged from P’s story included: voluntary HIV testing; shock and indignation after a positive diagnosis; marriage as a risk factor of HIV infection; and mental health stressors of depression, blame and suicidal ideation living with HIV. This case also indicates that HIV can induce certain behavioural changes and medical illnesses. In addition, the importance of good patient–doctor relationship in treating HIV patients and assuring patient’s adherence to treatment are reported in this case. Issues related to depression, alcohol abuse and anxiety are also discussed.

The researcher observed that in the hospital P shared her room with another female patient. While visiting P, treatment and care was given to both roommates by a nurse. The weight and temperature of P was taken and her roommate had the wound on one of her legs treated. The researcher was surprised and noted that the nurse did not ask the researcher to leave the room in order to perform her duties. It was also observed that P went around the room bare-footed and explained that she needed to feel the coolness of the floor.

### P’s Background

P was 49 years old at the time of the interview and originates from... (name of country of origin withheld). She is a university graduate and was diagnosed HIV positive in 1987 at the age of 23 years. She is married and has a child. She contracted the disease through a monogamous relationship with her husband, who is also HIV positive. She is still in medical care and is one of the oldest living patients with HIV that participated in the study. P reported that the disease contributed to her decision not to have more children. At the time of the interview, she was undergoing alcohol disintoxication treatment. She suffered depression and resorted to alcohol, leading to alcohol addiction. P has other medical and mental health problems. She is currently unemployed and on disability benefits. She indicated that she will resume activities after her treatment. P disclosed her HIV-positive status to her parents and sisters and not to friends. P is lonely, apparently lacking social support, creating stressors such as depression and anxiety, and adopts behaviours that are detrimental to her physical and mental health.

## 4. Discussion

### What Her Story Says

This paper is grounded on the narrative of the longest living HIV patient who participated in a larger study on the experiences of sub-Saharan African migrant women living with HIV in Belgium, which was done between April 2014 and December 2015. The main aim of this paper was to describe a SSA migrant woman’s lived experience, and also to use the individual story to raise questions about the larger context after an HIV diagnosis. This illustrative case highlights relational risks, personal transformation, search for normality, depression, fear of stigma, societal gender norms, and support among SSA women living and aging with HIV infection. It is probably the first case to suggest that SSA HIV-positive migrant women in Belgium can live longer if free and accessible ART is adhered to, and they receive social support, etc.

This case illustrates the vulnerability and the precarious health-related environment of SSA women to HIV infection wherever they happen to be. This case also depicts that married and unmarried African women are often at high risk of HIV and also inform how HIV virus could spread not only because of cultural practices but also because of individual behaviour and response to everyday life situations. P’s story also indicates the psychological, social and economic challenges women face even when they are aware about HIV transmission and prevention.

Major themes that emerged from P’s story included voluntary HIV testing; shock and indignation after a positive diagnosis; marriage as a risk factor of HIV infection; and mental health stressors of depression, blame and suicidal ideation living with HIV.

Voluntary testing of HIV status as indicated in this illustrative case remains salient in the diagnosis, treatment and prevention of HIV, consistent with previous studies [44,45,46]. Had the participant not gone for testing, she might not have known that she was HIV-infected and taken measures to begin early medical care and follow-up. She might have presented late with an advanced stage of the illness that might have been difficult and costly to treat and control, as seen in previous research [30,47,48]. Moreover, her husband and aunt referred to in the narrative, also knew of their HIV status as a result of testing. The HIV-positive husband also started medical care and follow-up. Early diagnosis and start of medical treatment might have greatly contributed to participant’s survival. Many studies have reported missed diagnoses that have resulted in a number of deaths that could have been prevented with antiretroviral treatment [49,50]. However, we do not know if P’s husband already knew that he was HIV positive and used P’s aunt (a nurse) as a means to get P tested without questioning the rationale for HIV testing in a monogamous marriage that supposedly excludes third party relationship. Similar to previous studies, P deplored the fact that marriage, an institution that she had much desired, was the source of her illness [51,52,53].

HIV disclosure can be assisted. We documented similar stories where the other partner was encouraged to test for HIV as a disclosure strategy [31]. In the cited study, HIV testing and disclosure of HIV-positive status was made possible through mediation of a care provider. The patient had requested that her HIV-positive status be revealed to her partner when both received the results of the test. The care provider then provided counselling to both without revealing the already-known HIV status of the infected partner. The HIV-infected woman thus achieved disclosing her status and confirmed her wish that her partner remained uninfected because they habitually practiced unsafe sex. The participant also reported non-use of condoms, a behaviour common among most SSA migrant women with HIV.

Most SSA women are at risk because of the power dynamics in favour of men, and women’s subordinate position in the overall African culture [54]. This case also highlights the fact that a majority of women are not able to have a dialogue with sexual partners on sexual and reproductive health and HIV, as reported in previous research [55]. Most often, these women are “choice-disabled”, and have little choice as to when to have sex or use condoms when the need for protection is ubiquitous [56]. In relation to our study on the experiences of SSA migrant women with HIV in Belgium [57], we also found that some participants in the study who had attempted to question the risky behaviours of their partners were rejected or abandoned.

It was also interesting to note that HIV, like cancer, hypertension and cardiovascular diseases, carries the fatality of diagnosis [58,59,60]. However, despite progress in antiretroviral therapy, a positive HIV diagnosis engenders anxiety, depression and above all stigmatization that may enhance suicidal ideation because of the sexual connotation of the disease. A positive HIV diagnosis challenges the already precarious and subordinate role as custodians of future generation for most women. Some women in the larger study [57] saw themselves as carriers of death and doomed to die as evident in P’s recital of refusing to have another child for fear of transmitting a virus that would kill the child.

An important strength in using self-reporting or narrative is that it enabled the participant to break the silence and freely tell her story without restriction. This narrative unveiled the universal but divergent aspect of an African woman’s life with HIV. However, the paper does not suggest that all SSA migrant women share the same experience. We strongly believe that this narrative is not specific to SSA migrant women. It can be transferred to any region that also has high HIV prevalence among migrant communities. It serves as an illustrative case analysing vulnerabilities, challenges and coping with HIV on an everyday basis.

A narrative or self-reported story may have limitations inherent in qualitative case studies [61,62,63,64,65]. The narrative was that of the participant and the observations were made by the researcher. This story might have given a different perspective if a different research method had been used to check on reliability and reflectivity of the study [66,67,68]. Furthermore, it would be impossible for the research to be free of bias in studying women living with HIV, especially as HIV is an emotional topic and the researcher is an African woman, conversant with the cultural and gender power dynamics existing in most African countries. Listening and recording this story was painful and sad.

## 5. Conclusions

This illustrative case presents a summary of the experiences of living with HIV among SSA migrant women. We conclude that the SSA population should be encouraged to change behaviours that they considered natural and normal but that propagate HIV transmission. Culturally appropriate change strategies should be emphasized that will encourage women’s empowerment, reduce gender inequality and finally reduce HIV stigma and discrimination. Programs that can reach people whose culture and life circumstances put them at risk should be redesigned. More spaces should be created to allow women (and people) living with HIV to emerge and tell their stories that may impact interventions geared towards behavioural change in preventing new HIV infections.
